# Simultaneous saccharification and ethanologenic fermentation (SSF) of waste bread by an amylolytic *Parageobacillus thermoglucosidasius* strain TM333

**DOI:** 10.1186/s12934-022-01971-6

**Published:** 2022-11-28

**Authors:** Christopher C. Ibenegbu, David J. Leak

**Affiliations:** 1 Department of Biology & Biochemistry, University of Bath, Bath, BA2 7AY UK; 2 Chipsboard UK Ltd, Unit 5 Matrix Court, Middleton Grove, Leeds, LS11 5WB UK

**Keywords:** Bioethanol, Liquefaction, Maltooligosaccharides, Saccharification, Sandwich WB, Gelatinisation, Neopullulanase, Amyloglucosidase, ɑ-Amylase, ɑ-Glucosidase, Simultaneous saccharification and fermentation SSF), *Parageobacillus thermoglucosidasius*

## Abstract

The starch in waste bread (WB) from industrial sandwich production was directly converted to ethanol by an amylolytic, ethanologenic thermophile (*Parageobacillus thermoglucosidasius* strain TM333) under 5 different simultaneous saccharification and fermentation (SSF) regimes. Crude α-amylase from TM333 was used alone or in the presence of amyloglucosidase (AMG), a starch monomerizing enzyme used in industry, with/without prior gelatinisation/liquefaction treatments and *P. thermoglucosidasius* TM333 fermentation compared with *Saccharomyces cerevisiae* as a control. Results suggest that TM333 can ferment WB using SSF with yields of 94–100% of theoretical (based on all sugars in WB) in 48 h without the need for AMG addition or any form of heat pre-treatment. This indicates that TM333 can transport and ferment all of the malto-oligosaccharides generated by its α-amylase. In the yeast control experiments, addition of AMG together with the crude α-amylase was necessary for full fermentation over the same time period. This suggests that industrial fermentation of WB starch to bio-ethanol or other products using an enhanced amylolytic *P. thermoglucosidasius* strain could offer significant cost savings compared to alternatives requiring enzyme supplementation.

## Introduction

The global economy is in a process of transition away from the use of fossil carbon as a source of energy and chemicals towards the use of renewable energy and carbon sources. Depending on the sector, this may result in complete decarbonisation, but there will still be a requirement for carbon-based precursors in industry and where complete decarbonisation of the energy sector is impractical. In these instances, ensuring that the carbon originates from renewable plant-based/algal resources is critical to balance the inevitable end-of-life carbon emissions, and mitigate against the potential for global warming.

Microbial fermentation is well established as an effective way to produce useful molecules from plant-derived carbohydrates but, with low and intermediate value products, the cost of the carbon feedstock makes up a large proportion of the process cost [32, 33, 49, 61] and can render a process non-viable. The use of lignocellulose-derived carbohydrates for bio-ethanol production is a classic example where, despite much progress in cost-reduction, this is still not competitive with using starch as the source of carbohydrate [49], despite the fact that starch comprises only a small proportion of the plant biomass. Notwithstanding, the combination of metabolic and protein engineering enables us to consider producing new compounds to supplant fossil carbon-derived chemicals and also to consider different production hosts based on their process properties, including their ability to grow on cheaper substrates such as those found in food-waste.

In the UK, bread is one of the most wasted foods with bread wastes of up to 1.2 million tons annually [37]. A recent report showed that over 11.5 billion sandwiches are typically consumed in the UK annually when home and industrially produced sandwiches are combined [6, 18] and, prior to the Covid 19 pandemic, the sandwich industry was growing at a steady rate of 2% or 80 million sandwiches each year [29]. The waste bread (WB, primarily the heels/crusts) generated from sandwich production has also increased with an estimated 24 million slices of bread thrown away annually by UK households, while the sandwich industries discard about 17% of the total bread that enters the factory [57]. Most of this ends up in anaerobic digestion, or in animal feed formulations as a low value feed [57]. Bread contains 50–75% starch, 10–15% protein and 0.3 to 5% sugars [12] and is potentially an ideal low-cost fermentation substrate. Starch is generally comprised of 20–30% amylose and 70–80% amylopectin, and several studies have demonstrated the conversion of bread to bioethanol after high temperature gelatinisation, liquefaction and saccharification with combinations of industrial enzymes [16, 27, 28, 38, 46]. Conversion of WB into other compounds like lactic acid [43, 52], Omega-3 rich oil [56], baker’s yeast [4], biohydrogen [13], enzyme production [39] and aromatic compounds [10] has also been reported. Industrial scale fermentation of surplus bread into alcoholic beverage has also been demonstrated through the traditional starch to ethanol bioprocess in the UK and Sweden [54, 57]. However, the conventional starch fermentation bioprocess is still not very economical, due largely to the cost of the high temperature gelatinization and the multiple enzymes needed for conversion to glucose for organisms such as *Saccharomyces cerevisiae* to ferment, as it cannot directly utilize starch or its oligosaccharides [7, 19–21, 30, 40, 48, 62] with the exception of maltose. Various pH and temperature adjustments required during gelatinization, enzymatic liquefaction and pre-saccharification also add to the cost.

*Parageobacillus thermoglucosidasisus* NCIMB 11,955 encodes an α-amylase and maltose transporter in its genome and is naturally amylolytic. Recent genome sequence analysis suggests that it can also secrete a neopullulanase and probably transports branched malto-oligosaccharides, providing it with the complete arsenal of enzymatic activities to break down starch [31]. It has been engineered to create a homoethanologenic strain TM242 and its amylolytic capabilities have been enhanced by incorporation of a second amylase gene, derived from *G. stearothermophilus* into its genome, creating strain TM333 [2, 3, 8].

Using bio-ethanol production as an example, together with various pre-processing scenarios from traditional pretreatment to “self-sufficient” simultaneous saccharification and fermentation (SSF), in this study we have explored the potential of *P. thermoglucosidasius* TM333 to use WB as a fermentation substrate with *S. cerevisiae* as a comparator. Simultaneous saccharification and fermentation, in which polymeric/oligomeric substrates are hydrolysed and fermented at the same time [35, 44], can reduce total processing time (by removing the need for a separate saccharification step), reduce overflow metabolism which can occur when cells are grown in high concentrations of easily metabolized sugars, and allow transport and intracellular metabolism of oligomeric carbohydrates as they are produced, which is an advantageous features of *Parageobacillus/Geobacillus* spp. In “self-sufficient” SSF the enzymes added during the fermentation actually derive from the same organism as used in the fermentation process, so do not require extensive purification.

## Materials and methods

### Materials

Un-used heels from sandwich manufacture were obtained from Greencore, UK, dried at 40 °C in an oven for 24 h (to 91.5% oven dry solids) and stored in sealed bags. Pullulanase (PULL, E2412) and amyloglucosidase (AMG, A7095) were sourced from Sigma Aldrich UK, while *P. thermoglucosidasius* TM333, a thermophilic ethanologen derived from *P. thermoglucosidasius* TM242 [8] and incorporating an additional *amyS* gene from *G. stearothermophilus* DSM22 expressed from the *ldh* promoter was obtained from ReBio Ltd (Guildford, UK), formerly TMO Renewables. *S. cerevisiae* strain S228C was obtained from Dr A. Wheals, University of Bath. All other chemical reagents and carbohydrate standards for HPLC were analytical grade (> 99% purity) and purchased from Sigma Aldrich.

### Compositional analysis of WB

Carbohydrate compositional analysis of the WB was carried out following acid hydrolysis [53]. Oven dried bread was first milled to a powder in a coffee grinder. Triplicate samples (0.1 g, to 0.3 g) of the milled bread were hydrolyzed with 3 ml of 72% w/w H_2_SO_4_ in a 100 ml capped Durham bottle and incubated (Harrow Scientific LTD, UK) at 35 °C for 1 h with shaking at 100 rpm. Then the samples were diluted with 84 ml of Milli-Q water and autoclaved at 121 °C for 60 min, cooled to room temperature, and neutralized with calcium carbonate powder to pH 6–7. The samples were filtered through a 0.22 μm nylon membrane filter (Phenomenex) and stored at −20 °C prior to analysis by HPLC. A sugar recovery test was used to compensate for sugar loss/decomposition due to acid hydrolysis [53], while compensation for the moisture content was considered during calculations. Estimation of the original amount of starch before hydrolysis was made by multiplying the measured amount of glucose by 0.901. Errors represent ± the standard deviation of 3 independent samples.

### Αlpha-amylase and pullulanase production by TM333

A fresh inoculum of *P. thermoglucosidasius* TM333 was prepared from a -80 °C vial grown for 24 h on tryptone soya agar plates (TSA: 15 g casein peptone, 5 g soy peptone, 5 g sodium chloride and 15 g agar per liter). This was used to inoculate 50 mL of 2SPY medium (16 g soy peptone, 8 g yeast extract, and 5 g sodium chloride per liter), pH 7 in a 250 ml baffled flask and incubated aerobically at 60 °C with shaking at 250 rpm in an Innova 44 shaking incubator (New Brunswick Scientific Ltd, St Albans, UK) until the OD_600_ reached 7–8 (3-4 h). This culture was used at 2% inoculum to inoculate a 1% (w/w) soluble starch (or 1% (w/w) sterile WB) suspension prepared in 2SPY media. The media was either buffered with a combination of 1 M stock solutions of MOPS, BIS–TRIS and HEPES buffers (32.8 mM final concentration of each) or unbuffered and incubated at 60 °C, 250 rpm for 17 h. After fermentation, samples were centrifuged at 3200×*g*, 4 °C and 15 min, and the supernatant analyzed and used as the source of crude ɑ-amylase and pullulanase in all the SSF experiments.

### Αlpha-amylase and neopullulanase analysis

Crude α-amylase in the supernatant of TM333 was analyzed by the DNS method [5]. A 1% solution of soluble starch in citrate phosphate buffer of various pH was used as substrate, and 0.5 ml of diluted crude enzyme and 0.5 ml of the substrate in capped glass tubes were assayed in triplicate at various temperatures (35–100 °C) and pH (5.5–8.0) for 10 min. The reactions were stopped by the addition of 3mls of dinitrosalicylic acid (DNS) solution (10.6 g of 3, 5-Dinitrosalicylic acid, 19.8 g of NaOH, 306 g of sodium potassium tartrate, 4 g of phenol, 8.3 g sodium metabisulfite and 1416 mL distilled water) and boiled in a water bath for 5 min. The samples were cooled in water (4 °C), and 10 ml of distilled water was added to each tube, mixed by vortexing and the absorbance measured in a spectrophotometer (Jenway 6305) at 575 nm. Standard solutions of maltose were prepared in the same buffer and used to calculate the enzyme activity. In control reactions just the substrate was incubated, with the enzyme added after the addition of DNS. Spectrophotometer blanks contained reagents and DNS solution only. One unit of alpha-amylase activity is the amount of enzyme that released reducing sugars equivalent to 1 µmole of maltose per minute under the assay conditions. Neopullulanase activity was measured according to Duffner et al. [14]. A 1% pullulan solution in citrate phosphate buffer pH 7 was used as substrate. The reaction, including controls and blanks, was the same as that for α-amylase except that 1% pullulan was used as substrate, with maltose as standard. One unit of neopullulanase activity is the amount of enzyme that released reducing sugars equivalent to 1µmole of maltose per minute under the assay conditions. Errors represent ± the standard deviation of three independent assays made on a single biological sample.

### SSF ethanol fermentation by *P. thermoglucosidasius* TM333 and *Saccharomyces cerevisiae* from WB

The standard process for industrial bioethanol production from starchy biomass involves: (1) gelatinization at 100 °C, pH 6.5 in the presence of a thermostable amylase (typically 15 min), (2) liquefaction at 85 °C, pH 6.5 with further amylase addition (typically 60 min), (3) saccharification (typically 3 h) at 60 °C, pH 5.5, after the addition of amyloglucosidase (and pullulanase, if the economics allow), (4) yeast fermentation at 35 °C, pH 5.5 Therefore, this regime provided the starting point for the current SSF analysis using WB. The dried WB was initially milled in a blender (Kitchen Perfected Table Blender with Grinder, Lloytron, UK), wrapped in aluminum foil and sterilized dry in an autoclave at 121 °C, 15 min to eliminate microbial contaminants. The powdered bread was adjusted to 3.6% (w/w) dry solids with 2SPY media for bacterial fermentations or with YP media (15 g/L yeast extract and 8 g/l peptone) for yeast fermentations in 500 ml sterile Durham bottles.

Five SSF strategies were investigated, with strategies 1 to 5 involving a gradual simplification of the process. Strategies 1 and 2 involved gelatinization (100 °C, pH7.0) and liquefaction processes (85 °C, pH 7.0) in the presence of TM333 α-amylase (crude culture supernatant) prior to SSF. Strategy 3 involved only gelatinization before SSF, while 4 and 5 involved no pretreatment but addition of crude TM333 α-amylase with (SSF4) or without (SSF 5) commercial AMG supplementation. After each treatment/process the pH was adjusted to 5.5 prior to yeast fermentation, using 5 M H_3_PO_4_. No further pH adjustment was made for TM333 fermentations as the final pH remained between 6.85 and 7.0, but MOPS, BIS–TRIS and HEPES buffers were added as described in “Αlpha-amylase and pullulanase production by TM333” section for every TM333 fermentation. The inoculum of *P. thermoglucosidasius* TM333 was prepared as described for amylase production (“Αlpha-amylase and pullulanase production by TM333” section), but after the 24 h plate cultures, a further liquid culture was prepared by inoculating a loopful of the TSA plate culture into 50 ml 2SPY media in a 250 ml flask and incubated at 60 °C, with shaking at 200 rpm for 4 h (OD_600_ of 7–8).

Yeast inoculum was prepared from a −80 °C glycerol stock spread on a YPD agar plate (10 g yeast extract, 20 g peptone, 20 g glucose and 15 g/l agar), and grown for 24 h at 35 °C, then a loopful from the plate was used to inoculate 50 ml of liquid YPD in a 250 ml baffled shake flask and grown at 35 °C with shaking at 180 rpm. After 24 h, the yeast culture was diluted to an OD_600_ of 18 with sterile distilled water for use as inoculum for SSF fermentations. Inoculation of yeast or bacteria was at a ratio of 1:10 (v/v) inoculum: bread media in a 15-mL screw-capped tube containing 10 ml of a WB media and 5 drops of antifoam, added to minimize foaming, according to the experimental options listed below (taking into account the dilution factor, actual fermentations were at 3.0% w/w dry WB solids). In SSF 1 to 5, *S cerevisiae* was incubated at 35 °C and 180 rpm, while TM333 fermentations were at 60 °C with shaking at 250 rpm in triplicate for 24–92 h. A sterile needle plugged with sterile cotton wool for CO_2_ pressure removal was used to pierce the caps of the tubes and left in place during the fermentations [47]. Tubes were sacrificed periodically, centrifuged at 3200×*g* for 15 min at 4 °C, and the supernatant filtered through a 0.2 µm nylon membrane, then stored at − 20 °C for subsequent HPLC analysis. All experiments were carried out in triplicate without supplementation with Ca^2+^; a requisite for most calcium ion dependent α-amylases. Errors represent ± standard deviation of the 3 biological replicates.

The maximum and experimental theoretical ethanol yields were calculated as follows:

1 $${\text{Maximum theoretical ethanol yield }}{{\text{g}} / {\text{l}}} = {\text{ total monomeric sugars }}\left( {\text{g}}/\text{l} \right){\text{ added in WB}} \times 0.{51}$$

2 $${\text{Theoretical ethanol }}\left( {{\text{experimental}}} \right) \, {{\text{g}} / {\text{l}}} \, = {\text{ Total monomeric sugar used }}{{\text{g}} / {\text{l}}} \times 0.{51}.$$

#### SSF strategies

In SSF 1, milled bread was gelatinized at 100 °C together with 100 ml crude TM333 α-amylase per kg of bread in a 500 ml Durham capped bottle for 15 min in a shaking water bath. The starch in the bread was then liquefied by incubation with a further 100 ml of crude α-amylase per kg of bread at 85 °C for 1 h at 150 rpm in a shaking water bath (Grant OLS 200, Grant instruments, Cambridge, UK) with occasional manual mixing (vigorous shaking of the bread suspension) before SSF. During the subsequent SSF, amyloglucosidase and pullulanase (both at 0.65% v/w of bread) were added together with a 10% (v/v) inoculum.

During pre-treatment, 2 ml samples were centrifuged at 3200×*g*, 4 °C for 15 min, the supernatant filtered through 0.2 µm nylon filters and analyzed for total sugars (monomeric and polymeric) using the NREL method [53]. Fermentation samples were also analyzed by HPLC for sugars, ethanol and other metabolites as described below.

SSF 2 differed from SSF 1 by the omission of pullulanase during the fermentation. This allowed assessment of the use of AMG alone for the breakdown of the starch oligosaccharides generated by the α-amylase pretreatment. SSF 3 was effectively the same as SSF 2 but with the omission of the separate liquefaction and saccharification steps; however, α-amylase was added during the fermentation. After gelatinization the sample was cooled then inoculated with a 10% (v/v) inoculum of TM333 or yeast and 0.65% (v/w) of AMG plus 100 ml/kg of α-amylase before fermentation. The total α-amylase added was the same as in SSF 1 and 2. SSF 4 was the same as SSF 3 but omitted the gelatinization stage (200 ml of α-amylase + AMG was added during SSF), while SSF 5 was the same as SSF 4 but omitted the amyloglucosidase from the SSF. Thus, complete fermentation depended on activities generated by *S. cerevisiae* or *P. thermoglucosidasius* to degrade the dextrins generated by the α-amylase (200 ml crude enzyme per kg of WB, equivalent to 42,850 IU amylase/kg of bread).

### Analysis of sugars, oligosaccharides and metabolites by HPLC

After each treatment step or fermentation, samples were taken and centrifuged at 3200×*g* for 15 min. The supernatants were filtered through 0.2 µm nylon filter (Phenomenex Inc, Torrance, CA) prior to analysis. High Pressure Liquid Chromatography (Agilent HPLC 1200, Agilent Technologies, Santa Clara, CA) was used to analyze all samples using NREL methods [53]. For oligomeric sugar determinations, acid hydrolysis of the samples was carried out by adding 0.035 mL of 72% w/w H_2_SO_4_ to 1.0 mL of the filtered sample and autoclaving at 121°C for 60 min. The autoclaved samples were cooled slowly to room temperature before addition of calcium carbonate powder to neutralize the samples [53]. The samples were filtered as described earlier and analysed by HPLC. Fermentation samples were also filtered and analyzed for sugars, ethanol, and other metabolic products. Analytes were separated on a Phenomenex Rezex RHM Monosaccharide H + (8%) column (300 m × 7.8 mm, Phenomenex Inc, Torrance, CA) at 65 °C with 5 mM H_2_SO_4_ as the mobile phase at a flow rate of 0.6 mL/min. Detection was either by refractive index or UV absorbance (215 nm). The difference between the amount of sugars after and before acid hydrolysis was assumed to represent oligomeric/polymeric soluble sugars after correction for sugar recovery under acid hydrolysis conditions [53]. Preparations of pure compounds of > 99% (glucose, maltose, arabinose, xylose, glycerol and ethanol) were used as standards for quantification.

## Results and discussions

### Compositional analysis of waste sandwich bread

The compositions of the WB used in this study are shown in Table 1. This accords with [45] who reported that WB contained 68.9% starch. Sanchez et al. [52] and [12] also reported that bread contains between 55.3 and 83.3% glucose equivalents. The moisture content of the mixed bread, made up of a combination of 22 different types of wholemeal and white bread, was 35% ± 13.5. After oven drying at 45 °C for 24 h, the moisture content was reduced to 8.5% ± 0.8 (w/w) to prevent spoilage during storage. The total carbohydrate content of the mixed WB (Table 1) was 70.58% starch (all of the glucose was assumed to derive from starch) with a small amount of arabinoxylan (2.26%) derived mainly from the wholemeal variety. The parent strain of TM333 can ferment hemicellulose-derived sugars (such as xylose and arabinose) and associated short chain oligosaccharides but does not encode a secreted xylanase or arabinanase [31].

**Table 1:** Carbohydrate composition (% w/w) of WB used in SSF

Type of bread	(Glucose)*	(Xylose)	(Arabinose)
Wholemeal bread	67.8 ± 2.8	1.2 ± 0.13	0.5 ± 0.02
White bread	75.0 ± 1.9	0.3 ± 0.05	0.01 ± 0.00
Mixed bread	70.6 ± 2.3	0.8 ± 0.11	1.5 ± 0.12

* (monomer) refers to the composition derived after hydrolysis, corrected to polymeric composition (starch and arabinoxylan) to account for the addition of water during hydrolysis

### Alpha-amylase and neopullulanase production by TM333

The crude α-amylase secreted by TM333 into the culture supernatant after 17 h of fermentation had a maximum activity of 428.5 U/ml at pH 7 and 85 °C but retained some activity at pH 5.5 and 35 °C (yeast fermentation condition), and also at the *P. thermoglucosidasius* TM333 optimal fermentation conditions of pH7 and 60 °C (Table 2). Malhotra et al. [34] reported that maximum Ca^+^-independent α-amylase production by thermophilic *B. thermooleovoran*s NP54, using a 2% inoculum was between 12 and 16 h, similar to the 17 h optimum fermentation time reported here.

**Table 2:** Alpha amylase activities of the crude enzyme from *P. thermoglucosidasius* TM333 under various assay conditions similar to SSF conditions used in this study

pH	Temperature °C	Activity *U/ml	Condition used
7.0	60	195.5 ± 3.5	*P. thermoglucosidasius* fermentation
7.0	85	428.5 ± 7.2	Liquefaction
7.0	100	8.1 ± 0.6	Gelatinisation
5.5	35	29.2 ± 1.4	Yeast fermentation

One unit of α-amylase is the amount of enzyme that released reducing sugars equivalent to 1 µmole of maltose per minute under the assay conditions.

**Fig. 1:**
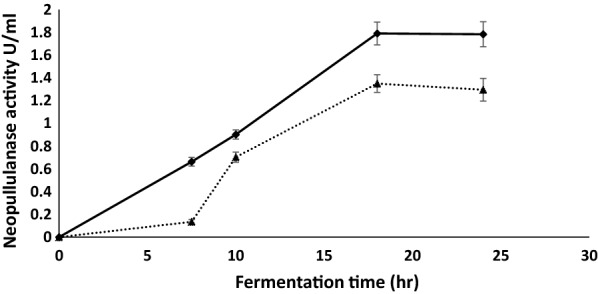
Comparison of neopullulanase production from TM333 grown on soluble starch (dotted line) and WB (solid line) assayed at 60 °C and pH 7.0. * One unit of neopullulanase is the amount of enzyme that released reducing sugars equivalent to 1µmole of maltose per minute under the assay conditions

Endoamylases are known to produce various chain lengths of linear and branched oligosaccharides from starch by acting on α-1–4, or α-1–6 glucose linkages [23]. In addition to α-amylase, low levels of neopullulanase activity (1.35 to 1.79 U/ml) were also measured in the culture supernatant produced either from soluble starch or WB at 18 h to 24 h (Fig. 1). Neopullulanase and pullulanase are known to increase the hydrolysis of starch by α-amylase, especially via the removal of α 1–6 branching or cleavage of dextrin to form panose, but they are expensive enzymes that are produced in low titers and are rarely added to starch hydrolysis for economic reasons. The use of pullulanases can reduce the requirement for glucoamylase by ~ 50% and can reduce the total reaction time of industrial starch conversion. A higher neopullulanase titre was produced on WB (1.79 U/ml) than on soluble starch (1.35 U/ml), suggesting that this might be a cheaper substrate for both α-amylase and neopullulanase production compared with purified soluble starch. The presence of protein, various vitamins and minerals in WB compared with purified soluble starch might have resulted in better enzyme production from WB. A DHSS [11] report on the nutritional aspects of bread and flour established that UK breads contain between 8 to 9% protein, several essential amino acids, fats and fatty acids, minerals, vitamins and trace elements which might enhance microbial growth and enzyme production.

### WB processing for SSF 1 to 5

**Fig. 2:**
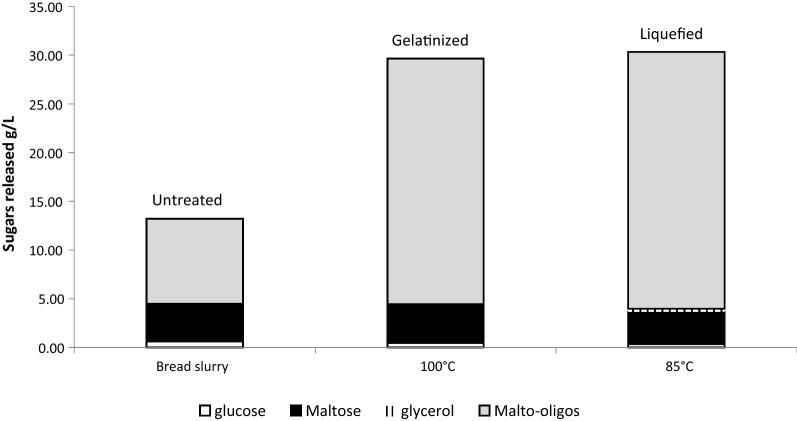
TM333 fermentation media produced from 3.6% (w/w) WB in SPY media after 100 °C and further 85 °C treatment with crude TM333 alpha-amylase. For clarity, the oligomers are shown as glucose equivalents. Malto-oligos = malto-oligosaccharides

Figure 2 shows the sugar composition of the WB in media without pretreatment, after gelatinization and after both gelatinization and liquefaction in SPY media; the same treatments done in YP media gave virtually identical results. The untreated WB suspended in media shows the presence of soluble malto-oligosaccharides and maltose with trace amounts of glucose (glucose < 0.5 g/l, maltose 3.6 to 3.9 g/l, malto-oligosaccharides 8.7–9.2 g/l) due to the presence of partially hydrolyzed starch. The low levels of glucose and maltose after 1 h of liquefaction suggest that TM333 α-amylase is not a maltogenic α-amylase. Van Zyl et al. [60], showed that α-amylases hydrolyze the internal α-1,4-bonds of starch amylose and amylopectin randomly, leading to the production of maltodextrins with a length of 10 to 20 glucose residues as major products, as well as smaller amounts of maltose and free glucose. These dextrins are known to be water soluble [23, 59]. In a control experiment (results not shown), WB without autoclave treatment was processed in the same way, in the presence of 0.2% (w/v) sodium azide to reduce microbial growth. The sugar profiles were similar to those in Fig. 2 indicating that the autoclave treatment did not contribute to starch hydrolysis in the WB.

### SSF1: SSF of gelatinized and liquefied WB with amyloglucosidase + pullulanase.

**Fig. 3:**
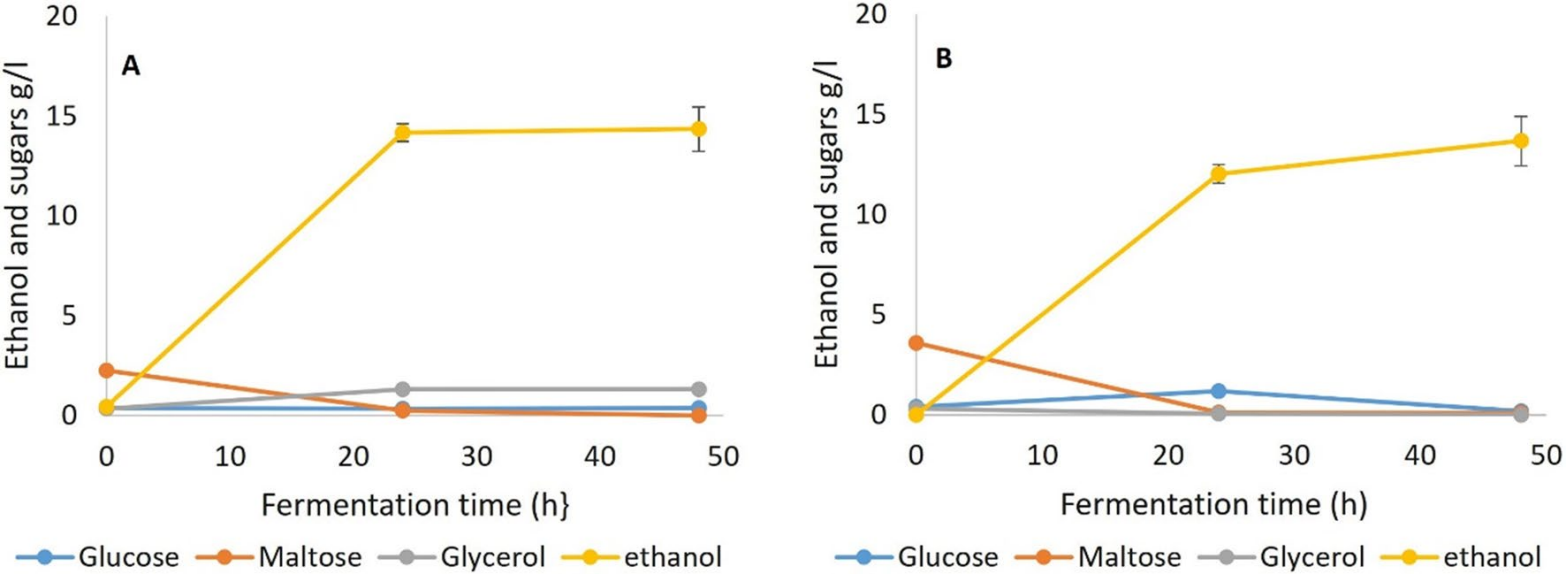
Fermentation of gelatinized and liquefied WB with amyloglucosidase + pullulanase both added during fermentation (SSF 1), **A** Yeast at 35 °C and pH5.5, **B** TM333 at 60 °C and pH7

Fermentation results for SSF 1 with amyloglucosidase and pullulanase included in the process are shown in Fig. 3. As described, the bread samples had been pre-gelatinized and liquefied with α-amylase from TM333 to yield mainly maltodextrins (Fig. 2). While AMG is important for the generation of glucose from straight chain malto-oligosaccharides, pullulanase attacks the 1,6 linkages, debranching the dextrins and also prevents the reverse reaction of glucose condensation to maltose or isomaltose, a process known to be catalysed by amyloglucosidase [36, 50]. The fermentation results show that complete fermentation of the WB was possible with yeast or TM333 at yields of > 98% of the maximum theoretical from all WB carbohydrates. Thus, the malto-oligosaccharides in the pretreated bread (Fig. 2) were being rapidly converted to maltose or glucose by the amyloglucosidase and/or pullulanase in combination with the remaining crude TM333 α-amylase under both sets of culture conditions.

### SSF 2: SSF of gelatinized and liquefied WB with amyloglucosidase added during fermentation

**Fig. 4:**
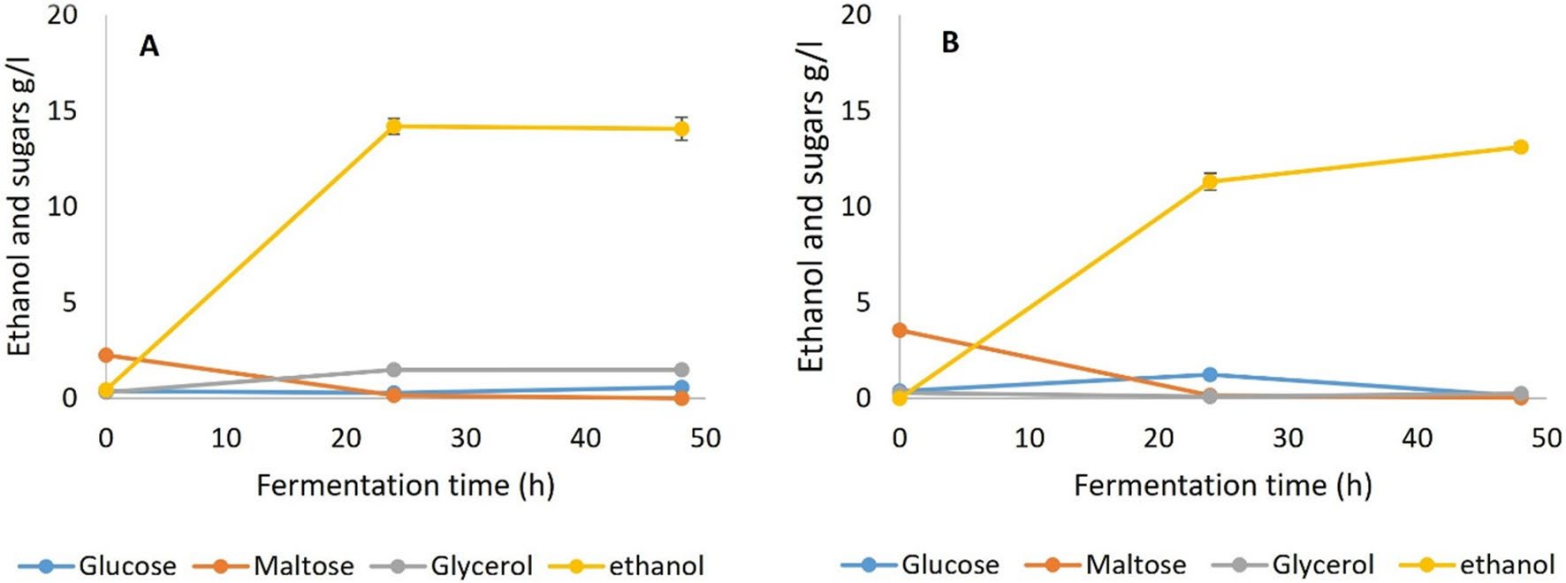
Fermentation of gelatinized and liquefied WB with amyloglucosidase supplementation during fermentation (SSF 2), **A** Yeast at 35 °C and pH5.5, **B** TM333 at 60 °C and pH7

In SSF 2 the debranching pullulanase was omitted from the SSF, but otherwise the conditions were the same as SSF 1. Fermentation results (Fig. 4) show that this SSF generated similarly high yields (> 98% of theoretical) for both yeast and TM333, indicating that the combination of TM333 α-amylase and AMG alone was sufficient to hydrolyse all of the malto-oligosaccharides to maltose or glucose, even at the sub-optimal temperature of 35 °C and pH5.5, with a fermentation profile similar to that in SSF 1. The α 1,6 debranching activities of AMG appeared to be sufficient to obviate the need for pullulanase, although it is not clear whether the low neopullulanase activity in the crude amylase might have been beneficial.

### SSF 3: SSF of gelatinized WB with amyloglucosidase added during fermentation

**Fig. 5:**
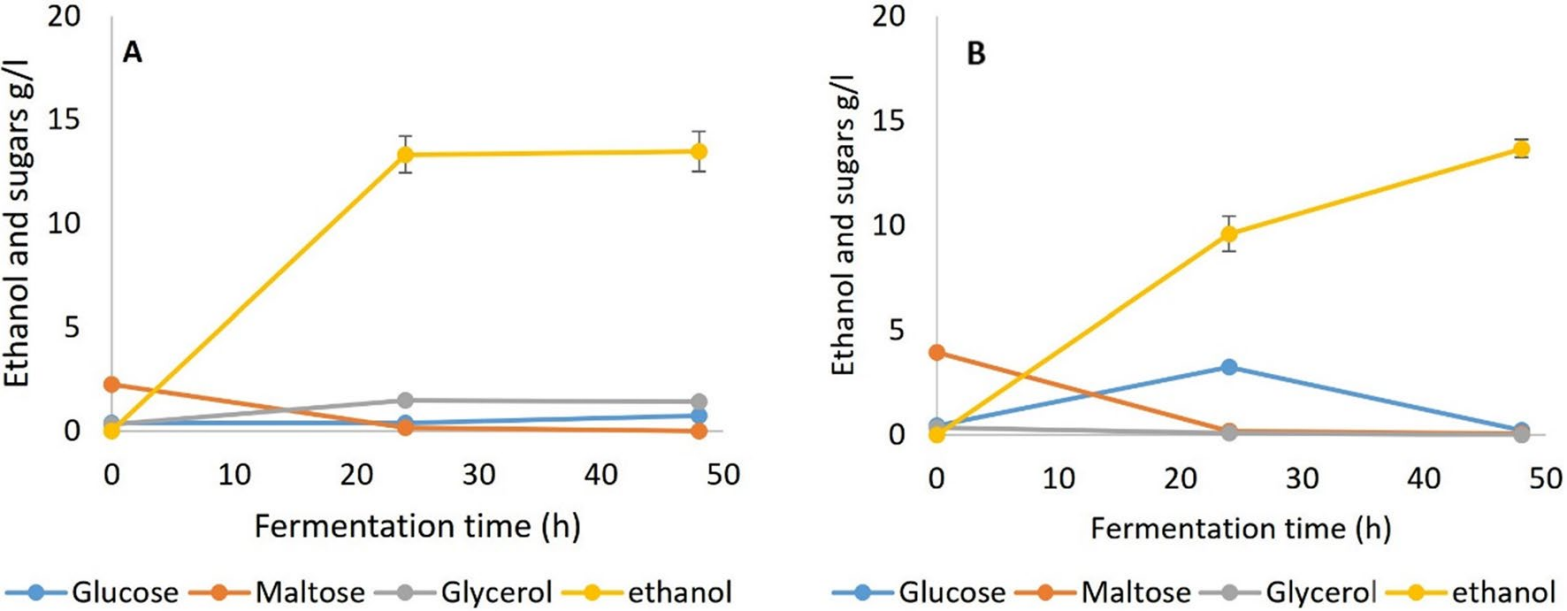
Fermentation of gelatinized WB with amyloglucosidase supplementation during fermentation (SSF 3), **A** Yeast at 35 °C and pH5.5, **B** TM333 at 60 °C and pH7

SSF 3 differed from 1 and 2 by not going through a liquefaction process after the initial gelatinization. The compositions of the SSF3 media prior to fermentation (Figs. 1 and 2) suggested that much of the initial α-amylase activity occurred during gelatinization, mainly producing malto-oligosaccharides with a small amount of maltose and less than 0.5% of glucose. Fermentation results (Fig. 5 and Table 2) showed that both TM333 and yeast fully fermented this media with yields of > 98% of theoretical. This shows that, with the quantities of α-amylase added, there was no need for liquefaction and pre-saccharification before fermentation when the crude enzyme of TM333 was added for both yeast and TM333 fermentations. However, TM333 fermentation was slower than seen in SSF 1 and 2 and there was a transient increase in glucose accumulation in the media, suggesting that the amyloglucosidase activity exceeded the capacity of cells to grow on the resulting glucose.

### SSF 4; SSF of untreated WB with crude α-amylase and amyloglucosidase added during fermentation

**Fig. 6:**
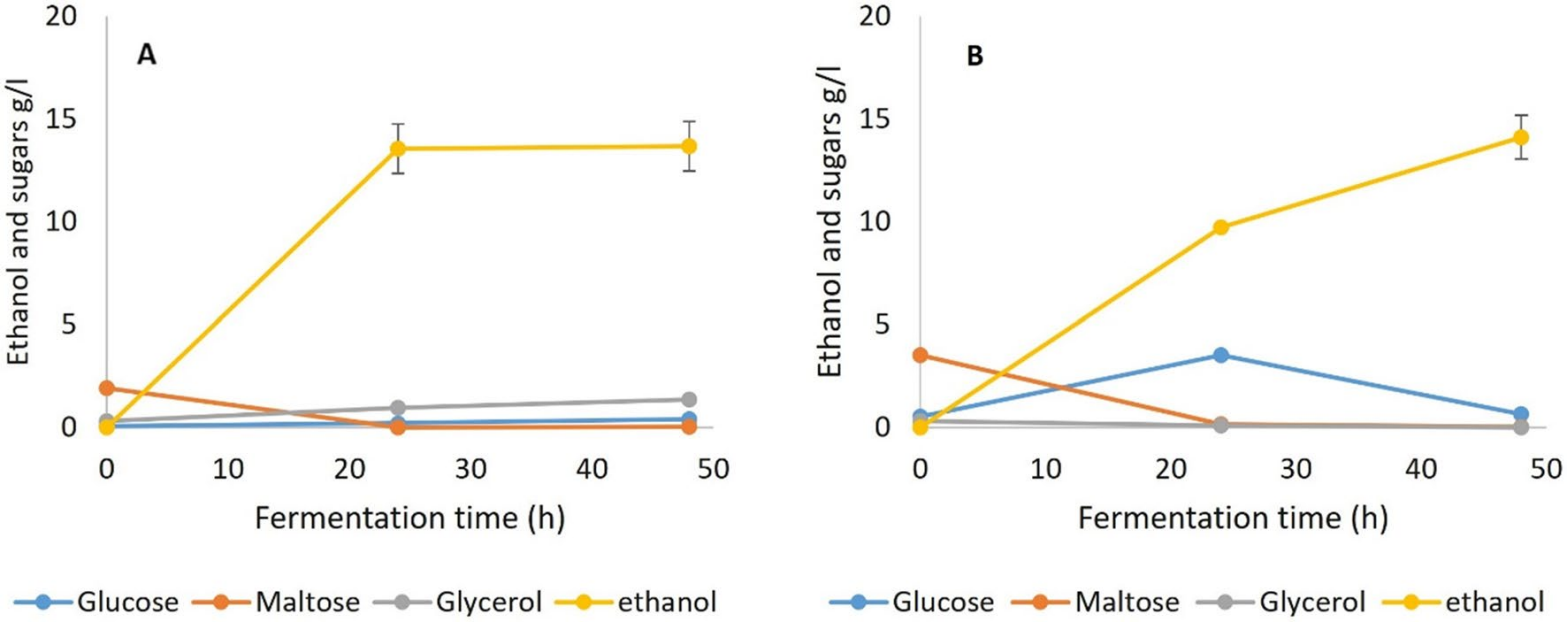
Fermentation of untreated WB with amyloglucosidase supplementation during fermentation (SSF 4), **A** Yeast at 35 °C and pH5.5, **B** TM333 at 60 °C and pH7

Given that some maltose and free glucose was present in the autoclaved breadcrumbs, this should allow initial growth of both organisms while supplemented enzymes hydrolyse the oligosaccharides and any residual unhydrolysed starch. Therefore, in SSF 4 untreated WB without gelatinization or liquefaction was used as the substrate and α-amylase and amyloglucosidase were added to the fermentation media (Fig. 6). Both yeast and TM333 were still able to fully ferment the WB in 24-48 h with > 98% of the maximum theoretical yield with comparable results to SSF 1 to 3. The fermentation profile for TM333 was similar to that of SSF 3, with a spike of glucose at 24 h.

The ability to degrade the residual non-hydrolysed starch within 24 h shows that the synergistic activity of the α-amylase and amyloglucosidase, even under the unfavorable conditions of 35 °C and pH5.5 was high, and that the autoclaved, but otherwise particulate starch was accessible to the TM333 amylase. It is, therefore, surprising that TM333 grew marginally more slowly under these conditions than in SSF 2. Given that there was glucose accumulating after 24 h, this suggests that intermediates in the breakdown of the polymeric starch were actually reducing the rate of growth, possibly by directly or indirectly (via regulation) affecting the glucose transporter. Given that *Parageobacillus* spp are known to be capable of transporting complex oligosaccharides this could be an example of evolutionary adaptation leading to impairment of growth under an artificial abundance of monosaccharide. Nevertheless, the cost savings in removing a pre-hydrolysis step and using a one-pot SSF should outweigh any marginal reduction in productivity. Furthermore, this effect might be resolved by optimising the balance of enzyme activity and nutrient uptake rate of the cells. The only comparative work on SSF of WB was by [45, 46], whereby a commercial enzyme cocktail (α-amylase and amyloglucosidase plus a protease) were used in combination with several types of pretreatment including heat, microwave, ultrasound (Table 3), but yields were still lower than those from our current work with only crude α-amylase.

**Table 3:** Comparison with previous reports of bread to ethanol process including SSF and SHF

Bread type	Treatment/fermentation conditions	Bread concentration % w/w	**Ethanol yield per kg of bread @ 48 h	Enzymes used	References
Waste wheat-rye bread	SSF (slurried with enzymes at 35 °C prior to fermentation)	15	333.31 (*354.36)	α-Amylase and glucoamylase, plus protease	[45]
Waste wheat-rye bread	SSF (slurried at 45 °C for 20 min prior to fermentation)	15	373.15 (*398.4)	α-Amylase, β-glucanase, pentosanase, cellulase, plus protease	[45]
Waste wheat-rye bread	SSF of liquefied WB	30	416–425	α-Amylase, amyloglucosidase plus protease	[46]
Waste wheat-rye bread	SSF post microwave pretreatment	15	375.5 (*384.6)	α-Amylase and glucoamylase, plus protease	[45]
Waste wheat-rye bread	SSF post Ultrasonic pretreatment	15	365.1 (*366.8)	α-Amylase and glucoamylase, plus protease	[45]
Waste wheat-rye bread	Separate hydrolysis and fermentation (SHF) standard process	15	378.7 (*386.0)	STARGEN 002 (α-amylase and glucoamylase) plus protease	[45]
mixture of wheat and buckwheat flours	SHF standard process	16	303.2–408.1 (67 h)	α-Amylase plus a glucoamylase	[1]
Sandwich WB	Yeast SSF of gelatinized and/liquefied WB	3	456–478.4 (*466–474.3)	α-Amylase plus a glucoamylase	This work
Sandwich WB	Yeast SSF of untreated WB	3	448.9 (*459.9)	α-Amylase plus a glucoamylase	This work
Sandwich WB	Thermophilic bacteria SSF of untreated WB	3	437.1 (*446.8)	*P. thermoglucosidasius* TM333 crude α-amylase only	This work

### SSF 5: SSF of untreated WB with TM333 α-amylase alone

**Fig. 7:**
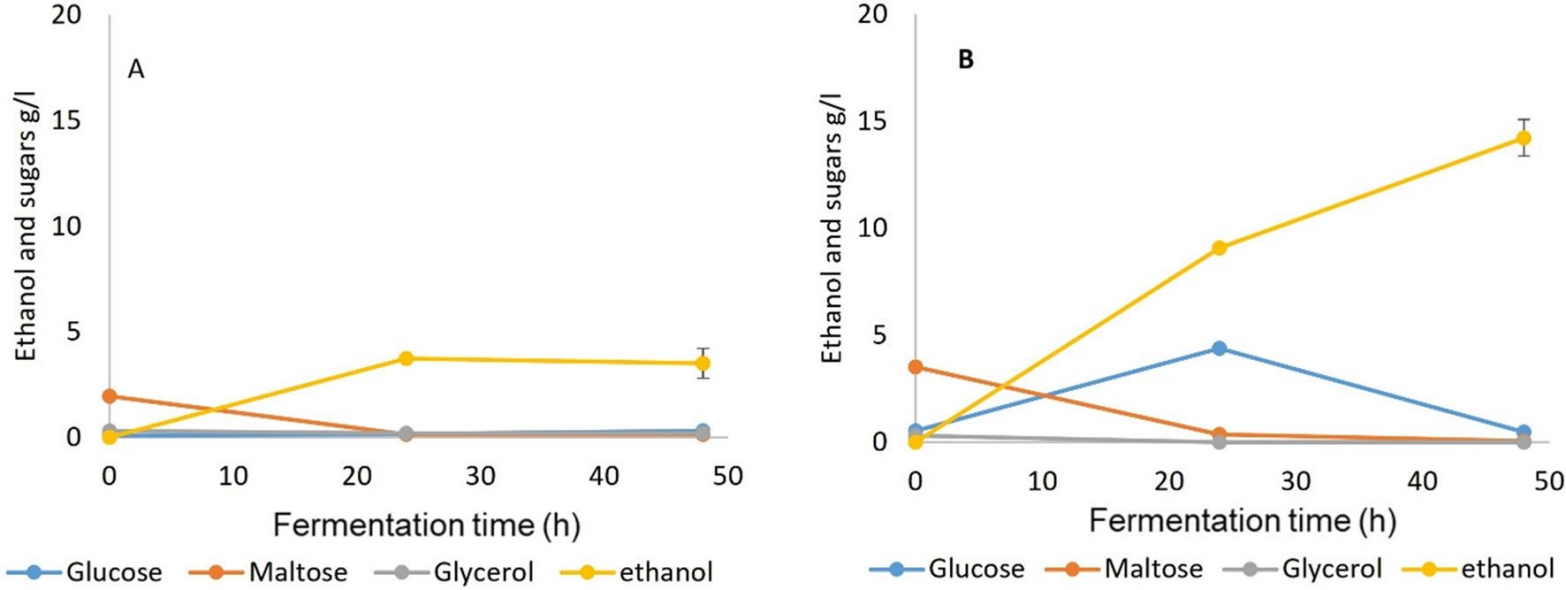
Fermentation of untreated WB with crude α-amylase as the sole enzyme supplement (SSF 5), **A** Yeast at 35 °C and pH5.5, **B** TM333 at 60 °C and pH7

As the TM333 α-amylase was clearly capable of degradation of the polymeric starch from WB, potentially down to maltose and branched limit dextrins the effect of a simple SSF on the autoclaved WB was examined in SSF 5. The lack of the saccharifying and 1,6-debranching amyloglucosidase was expected to reduce the amount of carbohydrate available for the yeast fermentation, but production of a neopullulanase by TM333 suggested that it should be able to import the product panose for further degradation. Given the results from SSF 4, the yeast fermentation in the presence of α-amylase alone was surprisingly poor (Fig. 7A), with less than 30% of the ethanol production seen in SSF 1 and 2. While this is more than can be accounted for by the maltose and glucose present in the untreated bread, suggesting that some maltose or glucose had been generated by the α-amylase, this is clearly fairly limited, with the saccharifying AMG being critical for good yeast SSF on WB. This is consistent with reports that maltose and glucose are minor products from the action of α-amylase on starch, [19, 21]. However, Fig. 7B shows that WB was fully fermented by TM333 in SSF with a native (actually a mixture or native and recombinant from the closely related *G. stearothermophilus*) α-amylase, with a very similar profile to that which was observed in SSF 4, suggesting that AMG had minimal contribution to the latter. The yield of ethanol using TM333 in this SSF was 94–96% of theoretical (14.24 g/l ethanol) based on the total sugars in WB, and seemingly even higher after 72-92 h fermentation (Table 3). With *S. cerevisiae*, however, the SSF with α-amylase alone gave a maximum ethanol yield of 26.8% of theoretical (3.74 g/l) based on total carbohydrate (Fig. 7A), with no increase at longer fermentation times (Table 3). The profile of the SSF 5 fermentation with TM333 was very similar to that of SSF 4 with a transient increase in glucose concentration after 24 h despite the lower ethanol production rate. The similarity suggests this was not the result of AMG activity but potentially the result of transglycosylation activity involving malto-oligosaccharides or neopullulanase activity, again suggesting that oligosaccharides were being metabolised in preference to glucose. Fermentation of other oligosaccharides by TM242, the parent strain of TM333 has been reported, including mannobiose and mannotriose from Palm kernel cake mannan [47], and also xylo-oligosaccharides and cellobiose [8, 31] with the help of its oligosaccharide active transport systems. Any regulatory effects of oligosaccharide utilization over monosaccharide uptake can potentially be modified through mutation.

### Comparison with previous studies

As far as we are aware, the only previous reports of WB SSF is the work of Pietrazak and Kawa-Ragieska et al. [45, 46] using SSF after various pretreatments and supplementing with commercial α-amylase, protease and amyloglucosidase. In some cases, cellulases, pentosanases and β-glucanases were also added to improve yields (Table 3). When adjusted to a common scale, these studies produced yields of 333–425 g ethanol per kg of WB [45, 46], with the highest yield only obtained by employing pre-liquefaction, in comparison with 437–447 g/kg produced by simple SSF with TM333 at 48 h and 72 h respectively in the current study.

The work of Pietrazak and Kawa-Ragieska et al. [45, 46] showed that initial high temperature gelatinization was beneficial for obtaining good ethanol yields, but this was less obvious in the current study. Two major starch transformations take place during the bread baking and storage process: firstly, crystalline starch is converted largely to its amorphous form during baking, while secondly during storage for > 2 days, the amylopectin units become more crystalline through retrogradation [25]. These two reactions are known to present a starchy material that normally requires high temperature gelatinization to be completely accessible to α-amylase. Additionally, amylose interacts with lipids to form complexes that are difficult for α-amylases to access and requires a liquefaction/melting temperature of around 105–116 °C [17, 22, 25] to improve accessibility, while amylose crystals are much more thermostable and melt at 150 °C [15]. Though fermentation was slightly faster in our experiments when gelatinization and liquefaction pre-treatment was incorporated, the yields without any treatment were comparable and not significantly different at P ≤ 0.05.

The SSF yields of ethanol obtained using the TM333 α-amylase, whether using *P thermoglucosidasius* or yeast (in combination with AMG) were higher in the current study than in previous reports (Table 3). Although this study used a lower bread concentration, Torabi et al. [58] showed that over a range of 9–16% (w/v), the concentration of WB did not affect the sugar and subsequent ethanol fermentation yields. Experimentally, it is difficult to demonstrate a *P thermoglucosidasius* SSF using higher concentrations of substrate without setting up a process of continuous ethanol removal, because ethanol toxicity starts to be observed above 2% (v/v). So, to confirm that the observations made using TM333 α-amylase hold true at higher loadings, SSF3 with yeast was repeated at WB loadings up to 25% (w/w).

**Fig. 8:**
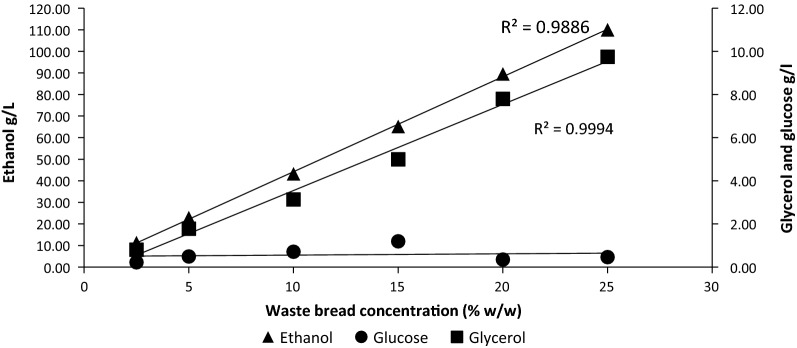
Ethanol production by *Saccharomyces cerevisiae* from various waste bread concentrations (2.5–25% w/w) after 24 h fermentation using the SSF3 protocol

Ethanol and glycerol yields after 24 h increased linearly over the waste bread concentrations tested (Fig. 8). The linearity (R^2^ = 0.989 for ethanol and 0.999 for glycerol) of the profiles indicates that there was no observed loss of enzyme performance or yeast fermentation inhibition as the waste bread concentrations increased from 3 to 27.5% during gelatinisation and from 2.5 to 25% during SSF ethanol production. The only notable effect of high substrate concentrations was that the concentration of glucose and maltose measured after gelatinization, reached a plateau at 15% (w/w) WB (Table 4). Glycerol production during yeast fermentation (Fig. 8) is a reaction to stress and acts as osmotic stabilizer for the yeast cells against high ethanol and acid concentrations produced during fermentation [51], but it diverts sugar away from ethanol production. The glycerol levels are consistent with those reported by [16] from 20% waste bread (43 g/kg of waste bread).

**Table 4:** Concentration of solubilized carbohydrates after gelatinization pre-treatment at various WB concentrations

Concentration of waste bread % g/g	Glucose g/l	Maltose g/l	Glycerol g/l	Malto-oligos g/l
2.5	0.34 ± 0.04	2.99 ± 0.28	0.07 ± 0.01	18.09 ± 0.46
5	0.81 ± 0.04	4.77 ± 0.26	0.18 ± 0.01	37.10 ± 1.5
10	1.23 ± 0.09	7.97 ± 1.2	0.29 ± 0.01	73.97 ± 1.61
15	1.49 ± 0.22	10.64 ± 0.6	0.39 ± 0.03	112.23 ± 1.72
20	0.15 ± 0.05	10.14 ± 0.66	0.42 ± 0.01	156.23 ± 1.29
25	0.96 ± 0.31	10.82 ± 1.0	0.57 ± 0.03	184.25 ± 3.0

With 25% (w/w) WB as substrate SSF3 produced approximately 11% (w/v) ethanol, equivalent to 440 g/kg WB, higher than virtually all previous reports and similar to that produced after liquefaction of the WB using α-amylase, amyloglucosidase and a protease [46]. This is also close to the yields obtained with both yeast and TM333 at lower substrate loadings. A possible explanation for these very high yields is that the crude α-amylase preparation from TM333 also contained a low activity of neopullulanase. Neopullulanase is a starch trimming enzyme with α-1–4 activity and some α-1–6 activity enabling conversion of highly branched amylopectin to the 3-sugar compound panose [24, 26] for direct transport into *P. thermoglucosidasius* or further hydrolysis by AMG for uptake into yeast. Indeed, Takata et al. have shown that neopullulanase can catalyse hydrolysis of α-(1–4)-glucosidic linkage, hydrolysis of α-(1–6)-glucosidic linkage, transglycosylation to form α-(1–4)-glucosidic linkage, and transglycosylation to form α-(1–6)-glucosidic linkages [55] and may be able to covert liquefied starch to malto-oligosaccharides without the presence of an α-amylase or amyloglucosidase.

It is notable that the disruption of the neopullulanase gene, *susA*, in *Bacteroides thetaiotaomicron* was found to reduce its rate of growth on starch by about 30% [9]. It is therefore possible that there was incomplete starch hydrolysis in most of the previous works shown in Table 3 due to the lack of a starch debranching enzyme. Interestingly, Novozymes [42] have recently launched an enzyme mixture (Spirizyme T) that contains both amyloglucosidase and pullulanase, with reported increase in both sugar and ethanol yields from starch. In a review on the use of pullulanases as debranching enzymes, Nisha and Satyanarayana [41] stated that the addition of pullulanase in starch hydrolysis would allow a reduction of glucoamylase usage by approximately 50% and reduce the total reaction time of an industrial starch conversion process. The possibility of using thermostable amylopullulanases in an economic one-step starch liquefaction and saccharification process, which replaces amylolytic enzymes thus resulting in the overall reduction in the cost of sugar production was also mentioned [41].

## Conclusions

A novel bioprocess, based on a self-sufficient simultaneous saccharification and fermentation of waste bread, using an amylolytic ethanologenic thermophile, *Parageobacillus thermoglucosidasius* TM333, to both generate the amylolytic enzyme cocktail and also carry out the fermentation, has been demonstrated. High ethanol yields (94–100% of the maximum theoretical based on all the carbohydrates in WB) were possible because TM333 can transport oligomeric sugars allowing it to ferment malto-oligosaccharides without the need for a starch monomerising enzyme. In addition, the production of both an amylase and a small amount of the debranching enzyme neopullulanase in the TM333 growth medium probably contributed to efficient substrate utilisation. Increasing the production of the native neopullulanase by recombinant methods would probably improve the fermentation rate. Unlike in previous studies, initial high temperature gelatinization of the starch in waste bread was not essential for the TM333 amylase to work. This, and dispensing with the need for an amyloglucosidase hydrolysis step, will significantly save on operational cost. Given that the amylase + neopullulanase cocktail is produced by *P. thermoglucosidasius* TM333 it may be sufficient to grow an inoculum on starch/WB and use a high (5–10%) inoculation rate for the main culture for this process to operate successfully. Alternatively, cell separation and enzyme concentration across a pair of cross-flow membranes might be necessary and would afford a greater degree of control.

The ability of the TM333 amylase cocktail to work at a wide range of pH and temperatures (35–100 °C) enabled demonstration of efficient mesophilic and thermophilic SSF of the starch in waste bread into ethanol, indicating that it may also have a wide application in several applications outside the biofuel industry. To the best of our knowledge, this is the first time it has been demonstrated that oligosaccharides (malto-oligosaccharides/malto-dextrins) generated by alpha-amylase catalysis of starch from WB during SSF can be effectively converted to ethanol by thermophilic bacteria at very high yields without the need for starch monomerising enzyme (amyloglucosidase).

## Data Availability

All data generated or analysed during this study are included in this published article. *Parageobacilllus thermoglucosidasius* TM333 is the property of ReBio Ltd.
